# Six versus four or five cycles of first‐line etoposide and platinum‐based chemotherapy combined with thoracic radiotherapy in patients with limited‐stage small‐cell lung cancer: A propensity score‐matched analysis of a prospective randomized trial

**DOI:** 10.1002/cam4.7215

**Published:** 2024-04-25

**Authors:** Tian‐tian Yu, Xiao Hu, Wei‐jian Liufu, Shao‐qing Niu, Hui‐min Lian, Hong‐lian Ma, Jin Wang, Yong Bao, Ming Chen, Fang Peng

**Affiliations:** ^1^ Department of Radiation Oncology The First Affiliated Hospital of Sun Yat‐Sen University Guangzhou China; ^2^ Zhejiang Cancer Hospital Hangzhou Institute of Medicine (HIM), Chinese Academy of Sciences Hangzhou China; ^3^ State Key Laboratory of Oncology in South China, Guangdong Key Laboratory of Nasopharyngeal Carcinoma Diagnosis and Therapy, Guangdong Provincial Clinical Research Center for Cancer Sun Yat‐Sen University Cancer Center Guangzhou China; ^4^ United Laboratory of Frontier Radiotherapy Technology of Sun Yat‐Sen University & Chinese Academy of Sciences Ion Medical Technology Co., Ltd Guangzhou China

**Keywords:** chemotherapy, concurrent chemoradiotherapy, cycles of chemotherapy, limited‐stage SCLC, radiotherapy

## Abstract

**Objectives:**

The recommended treatment for limited‐stage small‐cell lung cancer (LS‐SCLC) is a combination of thoracic radiotherapy (TRT) and etoposide plus cisplatin (EP) chemotherapy, typically administered over 4–6 cycles. Nonetheless, the optimal duration of chemotherapy is still not determined. This study aimed to compare the outcomes of patients with LS‐SCLC who received either 6 cycles or 4–5 cycles of EP chemotherapy combined with TRT.

**Materials and Methods:**

In this retrospective analysis, we utilized data from our prior prospective trial to analyze the outcomes of 265 LS‐SCLC patients who received 4–6 courses of EP combined with concurrent accelerated hyperfractionated TRT between 2002 and 2017. Patients were categorized into two groups depending on their number of chemotherapy cycles: 6 or 4–5 cycles. To assess overall survival (OS) and progression‐free survival (PFS), we employed the Kaplan–Meier method after conducting propensity score matching (PSM).

**Results:**

Among the 265 LS‐SCLC patients, 60 (22.6%) received 6 cycles of EP chemotherapy, while 205 (77.4%) underwent 4–5 cycles. Following PSM (53 patients for each group), the patients in the 6 cycles group exhibited a significant improvement in OS and PFS in comparison to those in the 4–5 cycles group [median OS: 29.8 months (95% confidence interval [CI], 23.6–53.1 months) vs. 22.7 months (95% CI, 20.8–29.1 months), respectively, *p* = 0.019; median PFS: 17.9 months (95% CI, 13.7–30.5 months) vs. 12.0 months (95% CI, 9.8–14.2 months), respectively, *p* = 0.006]. The two‐year and five‐year OS rates were 60.38% and 29.87% in the 6 cycles group, whereas 47.17% and 15.72% in the 4–5 cycles group, respectively.

**Conclusion:**

Patients diagnosed with LS‐SCLC who were treated with EP regimen chemotherapy combined with TRT exhibited notably enhanced survival when administered 6 cycles of chemotherapy, as compared to those who underwent only 4–5 cycles.

## 1 INTRODUCTION

Approximately 15% of lung cancer cases are attributed to small cell lung cancer, which is characterized by a rapid doubling time, a high growth fraction, and early development of widespread metastases.^1^ Chemo‐radiotherapy has demonstrated promising anti‐tumor activity in patients with limited‐stage small‐cell lung cancer (LS‐SCLC), achieving an impressive response rate (RR) of approximately 80%.^2^ However, median survival time (MST) for LS‐SCLC patients stands at 15–20 months.^1^ Despite the initially high RR of combined chemo‐radiotherapy, most patients suffer from local recurrence and/or distant metastasis within 2 years, indicating the important role of the systemic treatment.

The chemotherapy regimen, consisting of etoposide and cisplatin (EP), is widely employed as the primary treatment for LS‐SCLC^2^ and is recommended a maximum of 4–6 cycles by most guidelines. Maintenance or consolidation chemotherapy, in addition to this standard treatment, resulted in a minor prolongation of response duration, offering slight improvement in survival while elevating the risk of cumulative toxicity.^3^, ^4^ Nevertheless, the precise number of chemotherapy cycles for patients with LS‐SCLC remains uncertain.

In a prior prospective randomized clinical trial, we investigated thoracic radiotherapy (TRT) target volumes for LS‐SCLC and compared the outcomes between LS‐SCLC patients who underwent TRT to post‐induction chemotherapy and pre‐induction chemotherapy tumor extent.^5^, ^6^ Final results of the trial indicated that it was possible to limit TRT to the volume of the tumor after chemotherapy in LS‐SCLC patients. The chemotherapy delivered in this trial consisted of four to 6 cycles of EP.

Given the limited understanding of the optimal duration of EP‐based chemotherapy for LS‐SCLC, we conducted the current analysis, which involved performing a propensity score matching (PSM) analysis using individual patient data from the previous prospective trial. Our objectives were to provide additional clinical evidence regarding the outcomes and toxicity associated with administering 6 cycles compared to 4–5 cycles of the EP regimen in LS‐SCLC patients undergoing EP with concurrent TRT.

## 2 METHODS

### 2.1 Patients and eligibility criteria

Details of the prospective, randomized, noninferiority trial have been previously reported.^5^, ^6^ The trial followed the principles of the Declaration of Helsinki and Good Clinical Practice guidelines. It was approved by the clinical ethics committee of Sun Yat‐Sen University before study activation. This trial was registered with ClinicalTrials.gov under the identifier NCT01731548. The initial inclusion and exclusion criteria were described as previously reported.^5^, ^6^ For the clinical trial, the inclusion criteria included the following: age between 18 and 75 years inclusive; Karnofsky performance status (KPS) equal to or greater than 80; pathologic verification of SCLC; radiographic confirmation of limited‐stage; forced expiratory volume at 1 s (FEV1) equal to or greater than 1 liter; measurable lesions and sufficient liver, kidney, and bone marrow function; weight loss within 6 months prior to diagnosis less than 10%. Participants who had undergone thoracic radiotherapy, chemotherapy, or biotherapy before induction chemotherapy were excluded from this trial. Limited stage was defined based on the criteria of the Veterans Administration Lung Cancer Group. Individuals with a history of other malignant diseases, as well as those having contraindications for chemoradiotherapy were deemed ineligible.

**FIGURE 1:**
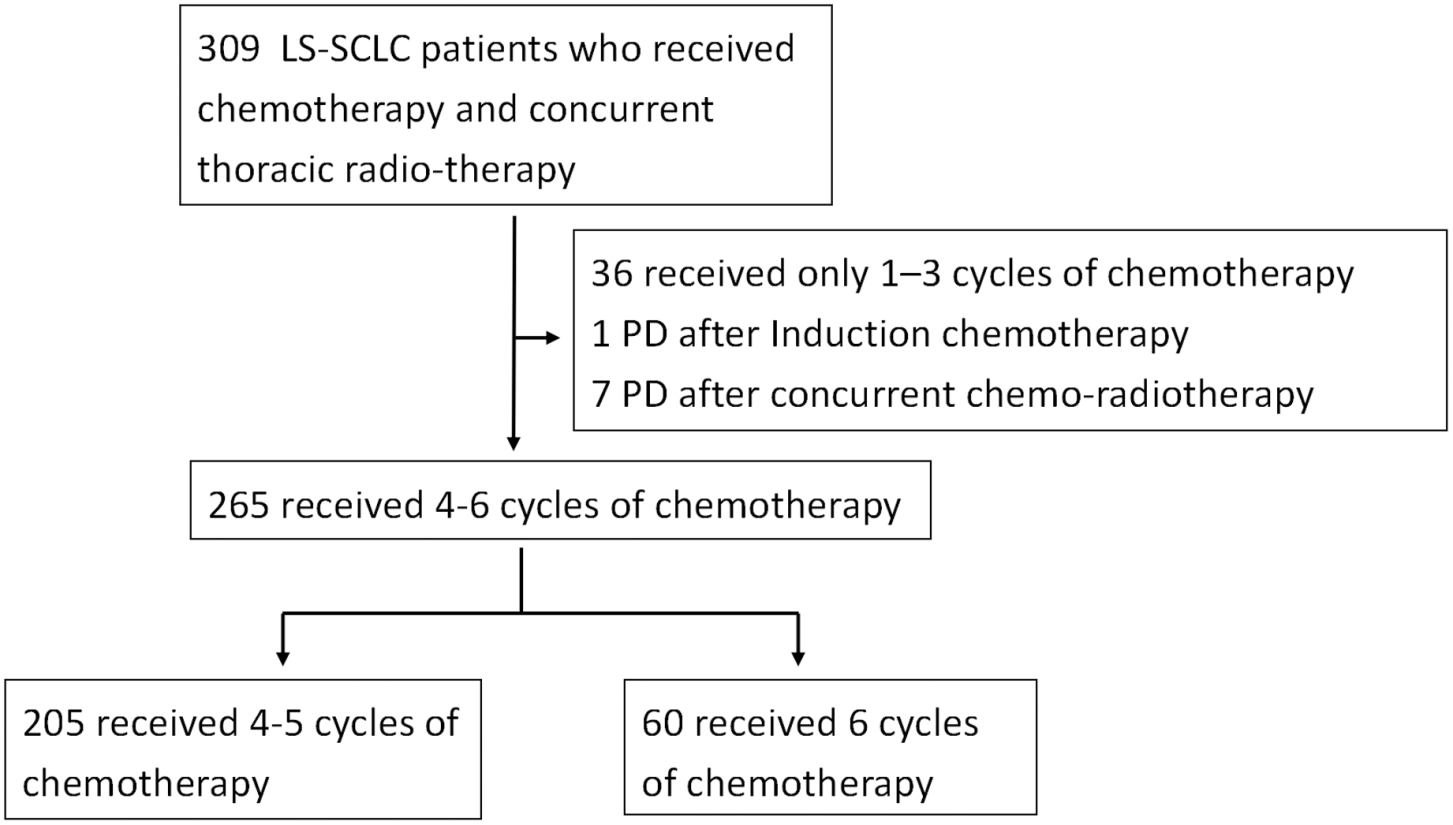
Patients' flow‐chart. LS‐SCLC, limited‐stage small‐cell lung cancer; PD, progressive disease.

Between June 2002 and January 2017, a cohort of 309 patients diagnosed with LS‐SCLC were eligible for the final analysis in the previous trial. For the current PSM analysis, 36 patients were excluded due to receiving three or fewer cycles of chemotherapy, and another 8 patients were excluded because they had disease progression after induction chemotherapy (IC) or concurrent chemo‐radiotherapy (CCRT). Ultimately, A total of 265 individuals diagnosed with LS‐SCLC were included in this study, all of whom underwent initial treatment consisting of 4–6 cycles of EP regimen chemotherapy and concurrently twice daily hyperfractionated TRT. Among the participants, there were 60 patients in the group receiving 6 cycles and 205 patients in the group receiving 4–5 cycles (Figure 1).

### 2.2 Treatment

Patients were treated with a chemotherapy regimen comprising of 4–6 cycles of EP. This protocol involved intravenous administration of etoposide (100 mg/m^2^ on days 1–3) and cisplatin (80 mg/m^2^ on day 1), with a 21‐day interval per cycle. All patients in this study underwent TRT, which was given concurrently with the third cycle of chemotherapy. The radiation plan consisted of delivering a total dose of 45 Gy over a period of 3 weeks, divided into 30 fractions at 1.5 Gy per fraction, twice daily. A linear accelerator utilizing photons ranging from 6 to 15 MV was used for treatment delivery. Following completion of IC, patients received TRT either before or after tumor extent evaluation.^5^ Those who achieved complete remission (CR) or partial response (PR) of tumor after the completion of CCRT were given prophylactic cranial irradiation (PCI). The total dose of PCI was 30 Gy administered in 15 fractions or 25 Gy in 10 fractions.

### 2.3 Follow‐up

Patients were reviewed at 4–6 weeks post‐treatment, followed by assessments every 3 months during the initial 2 years and subsequently every 6 months. Standard procedures included physical examinations along with regular thoracic and upper abdominal CT scans.

### 2.4 Propensity score matching (PSM)

To mitigate potential bias in this study, PSM was conducted between 6 cycle group and 4–5 cycle group. Significant clinical variables, including age, sex, weight loss, tumor stage, tumor type, concurrent radiotherapy, and PCI, were selected as matching factors during the PSM process. A match tolerance of 0.1 was set using a nearest neighbor approach with a ratio of 1:1 for matching.

### 2.5 Outcome measures and statistical analysis

The primary objective of this study was to examine whether the number of chemotherapy cycles administered to LS‐SCLC patients receiving EP with concurrent TRT had any impact on their overall survival (OS) and progression‐free survival (PFS). Patients were categorized into two groups depending on their number of chemotherapy cycles: either 6 or 4–5 cycles. Tumor responses were assessed using Response Evaluation Criteria in Solid Tumors Group version 1.1 (RECIST1.1), which categorized them as CR, PR, stable disease (SD), and progressive disease (PD). Adverse events were graded in accordance with the Common Terminology Criteria for Adverse Events version 3.0 (CTCAE 3.0). OS was calculated from the initiation of induction chemotherapy until the date of death or censored at last follow‐up. Likewise, PFS was determined from the start of induction chemotherapy to the first occurrence of objective PD, death, or censored at last follow‐up.

The statistical software package SPSS 26.0 (IBM, Somers, New York) was utilized for data analysis. To compare the distributions of continuous data, we used Wilcoxon and *t*‐tests. For comparing patient baseline characteristics and the incidence of toxicities between the two treatment groups, either *χ* ^2^ or Fisher's exact test was employed. The impact of the total number of chemotherapy cycles on both OS and PFS was evaluated using the Kaplan–Meier method and log‐rank statistic. Both univariate and multivariate analyses were conducted, incorporating the following parameters: age, sex, KPS, weight loss, tumor stage, tumor type, PET/CT examination, concurrent radiotherapy, PCI, tumor response after IC, tumor response after CCRT, and cycles of chemotherapy. The hazard risk of death, along with its 95% confidence interval (CI), was calculated. All *p* values were derived from a two‐sided test, and statistical significance was established at a threshold of *p* < 0.05. R version 4.0 was used to generate survival curve plots and forest plot.

## 3 RESULTS

### 3.1 Patient characteristics

Table 1 presents a summary of the patient characteristics. Among the 265 patients, the median age was 57 years (range: 34–75) in the 6 cycles group and 59 years (range: 34–75) in the 4–5 cycles group. The study mostly comprised males, constituting 83.8% of the participants. A significant proportion of patients exhibited a very good KPS (71.3%), and only a small percentage experienced weight loss (13.6%). Stage IIIa and IIIb patients constituted 29.8% and 63.0% of the total, respectively. PET/CT staging was applied to fewer than 20% of patients. All the patients achieved the planned 45Gy/30F TRT. There were some differences in baseline patient characteristics between the patients in the 4–5 cycles group and the 6 cycles group. A majority of patients underwent treatment with three‐dimensional conformal radiotherapy (3D‐CRT: 63.3%) in the 6 cycles group, whereas in the 4–5 cycles group, most patients received intensity‐modulated radiation therapy (IMRT: 54.6%). The patients who underwent PCI (183/265) received cranial CT or MRI before PCI. Of these, 164 patients underwent cranial MRI and 19 patients underwent cranial CT scanning. Approximately 56.7% of the patients were treated with PCI in the 6 cycles group, while 72.7% were in the 4–5 cycles group. The responses to induction chemotherapy for CR and PR were 10.0% and 56.7% in the 6 cycles group, while 1.0% and 89.3% in the 4–5 cycles group, respectively. As for responses to CCRT for CR and PR rates, in the 6 cycles group, the rates were 21.7% and 68.3%, respectively, while in the 4–5 cycles group, the rates were 32.7% and 64.9%, respectively.

**TABLE 1:** Patient characteristics.

Characteristic	Before propensity score matching no. (%) (*n* = 265)			After propensity score matching no. (%) (*n* = 106)		
	4–5 cycles	6 cycles	*p*‐Value	4–5 cycles	6 cycles	*p*‐Value
	(*n* = 205)	(*n* = 60)		(*n* = 53)	(*n* = 53)	
Age (years)			0.238			0.837
<60 years	117 (57.1%)	40 (66.7%)		36 (67.9%)	34 (64.2%)	
≥60 years	88 (42.9%)	20 (33.3%)		17 (32.1%)	19 (35.8%)	
Sex			0.373			0.759
Men	169 (82.4%)	53 (88.3%)		46 (86.8%)	48 (90.6%)	
Women	36 (17.6%)	7 (11.7%)		7 (13.2%)	5 (9.4%)	
KPS			1.000			0.829
80	59 (28.8%)	17 (28.3%)		14 (26.4%)	16 (30.2%)	
90	146 (71.2%)	43 (71.7%)		39 (73.6%)	37 (69.8%)	
Weight loss			0.256			1.000
<5%	174 (84.9%)	55 (91.7%)		47 (88.7%)	48 (90.6%)	
≥5%	31 (15.1%)	5 (8.3%)		6 (11.3%)	5 (9.43%)	
Tumor stage			0.668			0.841
I	3 (1.5%)	1 (1.7%)		0 (0.0%)	0 (0.0%)	
II	10 (4.9%)	5 (8.3%)		2 (3.8%)	4 (7.6%)	
IIIa	61 (29.8%)	18 (30.0%)		14 (26.4%)	14 (26.4%)	
IIIb	131 (63.9%)	36 (60.0%)		37 (69.8%)	35 (66.0%)	
Tumor type			0.086			0.656
Peripheral	44 (21.5%)	20 (33.3%)		12 (22.6%)	15 (28.3%)	
Central	161 (78.5%)	40 (66.7%)		41 (77.4%)	38 (71.7%)	
PET/CT examination			0.314			0.157
No	168 (82.0%)	45 (75.0%)		45 (84.9%)	38 (71.7%)	
Yes	37 (18.0%)	15 (25.0%)		8 (15.1%)	15 (28.3%)	
Concurrent radiotherapy			**<0.001**			1.000
2D‐CRT	7 (3.4%)	9 (15.0%)		4 (7.6%)	4 (7.6%)	
3D‐CRT	86 (42.0%)	38 (63.3%)		36 (67.9%)	36 (67.9%)	
IMRT	112 (54.6%)	13 (21.7%)		13 (24.5%)	13 (24.5%)	
PCI			**0.028**			1.000
No	56 (27.3%)	26 (43.3%)		20 (37.7%)	20 (37.7%)	
Yes	149 (72.7%)	34 (56.7%)		33 (62.3%)	33 (62.3%)	
Tumor response after IC			**<0.001**			**0.002**
CR	2 (1.0%)	6 (10.0%)		0 (0.0%)	4 (7.6%)	
PR	183 (89.3%)	34 (56.7%)		46 (86.8%)	31 (58.5%)	
SD	20 (9.8%)	20 (33.3%)		7 (13.2%)	18 (34.0%)	
Tumor response after CCRT			**0.019**			0.466
CR	67 (32.7%)	13 (21.7%)		14 (26.4%)	11 (20.8%)	
PR	133 (64.9%)	41 (68.3%)		37 (69.8%)	37 (69.8%)	
SD	5 (2.4%)	6 (10.0%)		2 (3.8%)	5 (9.4%)	

Abbreviations: 2D‐CRT, two‐dimensional radiotherapy; 3D‐CRT, three‐dimensional conformal radiotherapy; CCRT, concurrent chemo‐radiotherapy; CR, complete remission; IC, induction chemotherapy; IMRT, intensity‐modulated radiation therapy; KPS, Karnofsky performance score; PCI, prophylactic cranial irradiation; PR, partial response; SD, stable disease.

*Note*: Bold values are statistically significant.

After PSM, 53 patients were enrolled in each group. Both 36 (67.9%) patients in the 6 cycles group and 4–5 cycle group achieved 3D‐CRT. There were 33 (62.3%) patients in the 6 cycles group, and 33 (62.3%) patients in the 4–5 cycles group received PCI. The difference in concurrent radiotherapy and PCI between the groups was not statistically significant (*p* = 1.000). The responses to CCRT for CR and PR in the 6 cycles group were 11 (20.8%) and 37 (69.8%), while in the 4–5 cycles group were 14 (26.4%) and 37 (69.8%). The distinction between the two groups did not reach statistical significance (*p* = 0.466). However, the responses to induction chemotherapy for CR and PR were 4 (7.6%) and 31 (58.5%) in the 6 cycles group, in comparison to 0 (0.0%) and 46 (86.8%) in the 4–5 cycles group. The difference between the two groups remained statistically significant (*p* = 0.002).

### 3.2 Survival

**FIGURE 2:**
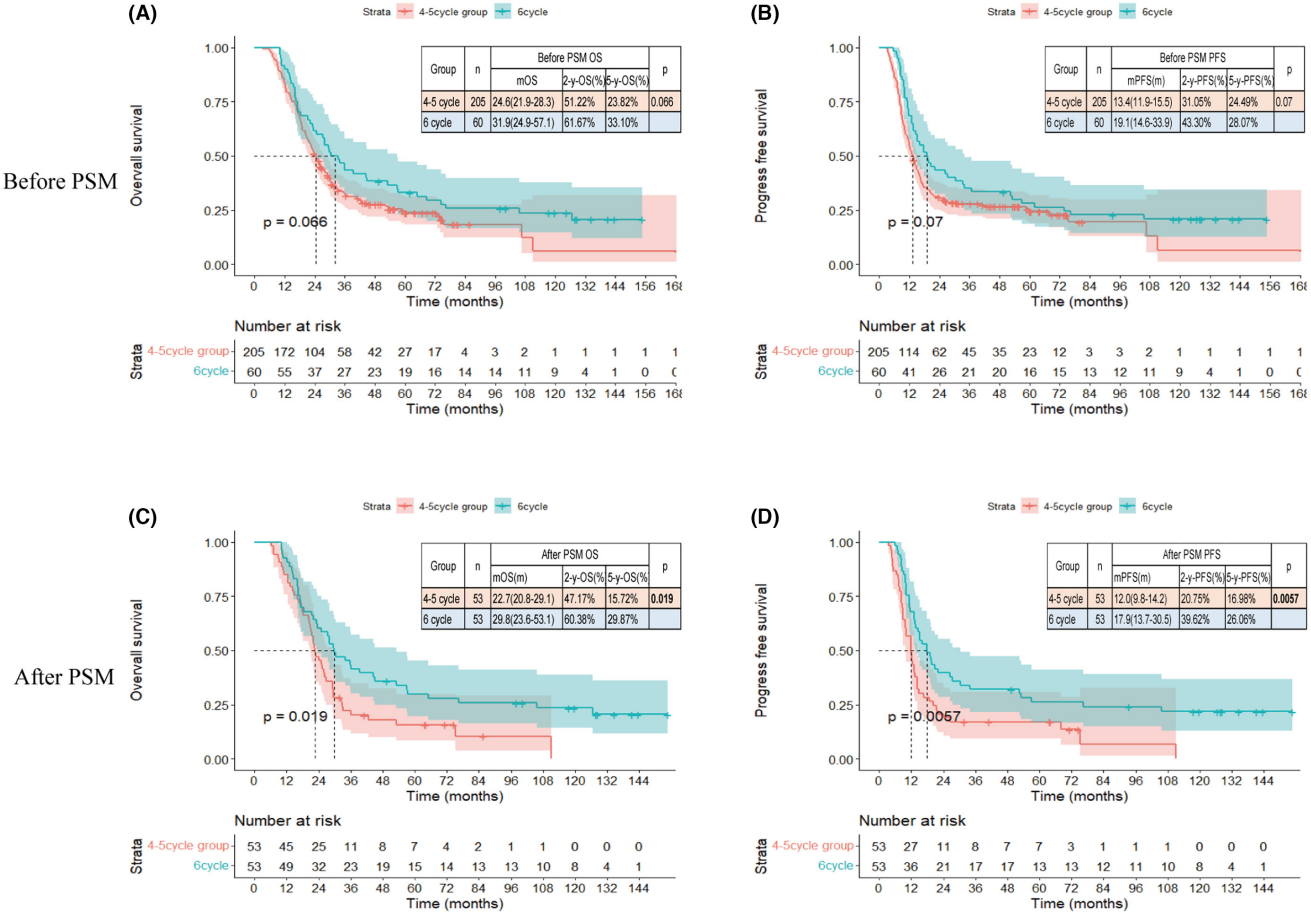
Overall survival (OS) and progression‐free survival (PFS) curves for patients with limited‐stage small‐cell lung cancer according to cycles of chemotherapy. (A) Before Propensity score matching (PSM), OS for patients in 4–5 cycles and 6 cycles group (*n* = 265, *p* = 0.066); (B) Before PSM, PFS for patients in 4–5 cycles and 6 cycles group (*n* = 265, *p* = 0.07). (C)After PSM, OS for patients in 4–5 cycles and 6 cycles group (*n* = 106, *p* = 0.019); (D)After PSM, PFS for patients in 4–5 cycles and 6 cycles group (*n* = 106, *p* = 0.0057).

All individuals were followed up until December 2018, and 200 (75.5%) patients had died by the end of the follow‐up period. Specifically, in the 6 cycles group, 46 patients (76.7%), while in the 4–5 cycles group, 154 patients (75.1%) had died. In the whole cohort, the median follow‐up time for the surviving patients was 41.8 months (range 10.7–169.0 months). During this period, the median OS and PFS were 26.0 months (95% confidence interval [CI], 23.1–29.5 months) and 14.2 months (95% CI, 12.9–16.5 months), respectively. The median OS was longer in the 6 cycles group at 31.9 months (95% CI, 24.9–57.1 months), compared to 24.6 months (95% CI, 21.9–28.3 months) in the 4–5 cycles group (hazard ratio [HR] 0.7 (0.519–1.020, *p* = 0.066), Figure 2A). Similarly, the 2‐year and 5‐year OS rates favored the 6 cycles group at 61.7% and 33.1%, respectively, compared to 51.2% and 23.8% in the 4–5 cycles group. Regarding PFS, the 6 cycles group also demonstrated a longer median PFS of 19.1 months (95% CI, 14.6–33.9 months), compared to 13.4 months (95% CI, 11.9–15.5 months) in the 4–5 cycles group (HR 0.7 (0.527–1.030), *p* = 0.07, Figure 2B). The results between the two groups differed numerically but not statistically.

After PSM, the median OS in both cohorts was 29.8 months (95% CI, 23.6–53.1 months) for 6 cycle group, 22.7 months (95% CI, 20.8–29.1 months) for 4–5 cycles group (HR 0.6 (0.388–0.924), *p* = 0.019, Figure 2C). The 2‐year and 5‐year OS rates for 6 cycles were 60.38% and 29.87%, and for 4–5 cycles, they were 47.17% and 15.72%. Following PSM, the 6 cycles group exhibited the median PFS of 17.9 months (95% CI, 13.7–30.5 months), in contrast to 12.0 months (95% CI, 9.8–14.2 months) for the 4–5 cycles group (HR 0.6 (0.359–0.844), *p* = 0.006, Figure 2D). The 2‐year PFS rates were 39.62% for the 6 cycles group and 20.75% for the 4–5 cycles group. And the 5‐year PFS rates were 26.06% for the 6 cycles group and 16.98% for the 4–5 cycles group.

### 3.3 Subgroup analysis

**FIGURE 3:**
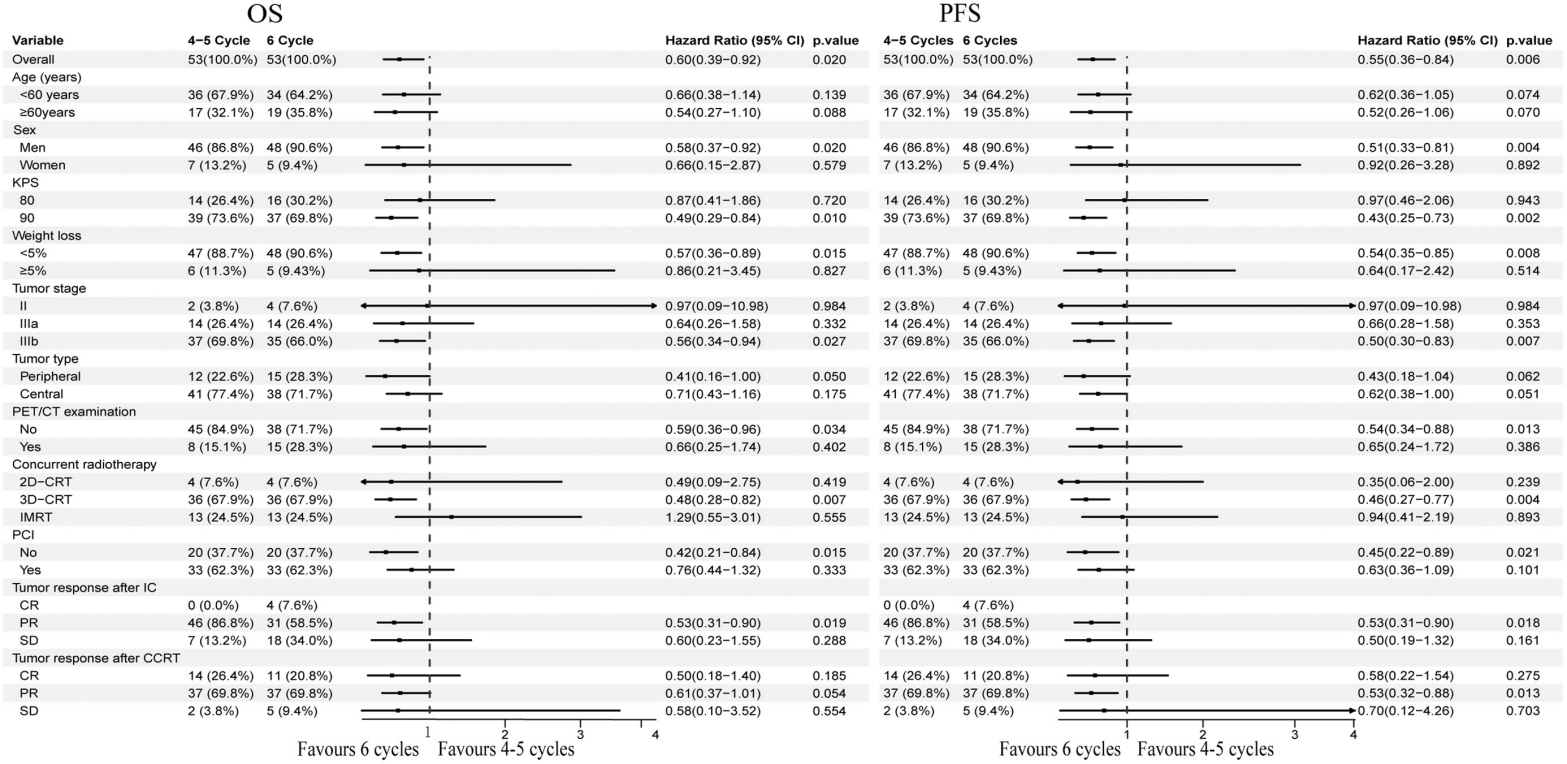
Treatment effect on overall survival (OS) and progress free survival (PFS) by subgroup. HR, hazard ratio.

Our results showed that 6 courses of chemotherapy were beneficial to improve survival both OS and PFS, with the hazard ratio (HR) values for OS and PFS were 0.60 (95% CI, 0.39–0.92, *p* = 0.020) and 0.55 (95% CI, 0.36–0.84, *p* = 0.006), respectively. In the subgroup of males, KPS = 90, weight loss <5%, tumor stage IIIb, without PET‐CT examination, using 3D‐CRT technique, without PCI, achieved PR after induction chemotherapy; patients received 6 courses of chemotherapy and achieved better survival (Figure 3). For further analysis, among the subgroup of patients who achieved PR after CCRT, those in the 6 cycles group had a longer OS and PFS compared to their counterparts in the 4–5 cycles group, and the HR values of OS and PFS were 0.61 (95% CI, 0.37–1.01, *p* = 0.054, close to 0.05) and 0.53 (95% CI, 0.32–0.88, *p* = 0.013) respectively. After PSM (35 patients for each group), it was observed that patients in the 6 cycles group sustained longer OS and PFS compared to those in the 4–5 cycles group (with median OS for 6 cycles vs. 4–5 cycles were 30.5 months (95% CI, 23.1–57.1 months) vs. 22.1 months (95% CI, 20.2–26.9 months), respectively, *p* = 0.022; median PFS for 6 cycles vs. 4–5 cycles were 17.9 (95% CI, 14.6–53.1 months) vs. 10.3 months (95% CI, 8.9–14.2 months), respectively, *p* = 0.007, Figure S1).

### 3.4 Prognostic factors

The results of univariate and multivariate analyses for OS and PFS were detailed in Tables 2 and 3. Univariate analysis indicated that KPS = 90, lower initial tumor stage, having undergone PCI, and having achieved CR after CCRT were significantly favorable prognostic factors for OS and PFS. Multivariate analysis (excluded if *p*‐value > 0.1) demonstrated having KPS = 90, lower initial tumor stage, having undergone PCI, having achieved CR after CCRT, and 6 cycles of chemotherapy were independent and favorable prognostic factors for OS. Meanwhile, PFS demonstrated significant associations with having a KPS of 90, lower initial tumor stage, having undergone PCI, and having undergone 6 cycles of chemotherapy.

**TABLE 2:** Univariate analysis for prognostic factors.

Variables	OS			PFS		
	HR	95% CI	*p*‐Value	HR	95% CI	*p*‐Value
Age (years)	1.2	0.899–1.580	0.224	1.0	0.765–1.340	0.933
(<60 vs. ≥60)						
Sex	0.9	0.652–1.380	0.775	1.2	0.811–1.660	0.417
(Men vs. Women)						
KPS	0.9	0.901–0.958	**<0.001**	0.9	0.905–0.961	**<0.001**
(80 vs. 90)						
Weight loss	1.0	0.683–1.560	0.879	1.0	0.670–1.510	0.980
(<5% vs. ≥5%)						
Tumor stage	1.4	1.090–1.700	**0.006**	1.4	1.100–1.710	**0.006**
(I‐IIIa vs. IIIb)						
Tumor type	1.1	0.825–1.580	0.425	1.0	0.725–1.370	0.992
(Peripheral vs. Central)						
PET/CT examination	0.8	0.590–1.210	0.365	0.8	0.547–1.130	0.188
(No vs. Yes)						
Concurrent radiotherapy	1.2	0.906–1.460	0.249	1.1	0.880–1.400	0.379
(2 or 3D‐CRT vs. IMRT)						
PCI	0.7	0.499–0.894	**0.007**	0.6	0.478–0.849	**0.002**
(No vs. Yes)						
Tumor response after IC	1.3	0.924–1.760	0.139	1.3	0.963–1.830	0.083
(CR or PR vs. SD)						
Tumor response after CCRT	1.5	1.140–1.950	**0.003**	1.4	1.050–1.780	**0.021**
(CR vs. PR or SD)						
Cycles of chemotherapy	0.7	0.519–1.020	0.067	0.7	0.527–1.030	0.070
(4–5 vs. 6)						

Abbreviations: 2D‐CRT, two‐dimensional radiotherapy; 3D‐CRT, three‐dimensional conformal radiotherapy; CCRT, concurrent chemo; CI, confidential interval; HR, hazard ratio; IC, induction chemotherapy; IMRT, intensity‐modulated radiation therapy; KPS, Karnofsky performance score; OS, overall survival; PCI, prophylactic cranial irradiation; PFS, progression free survival; VS, versus.

*Note*: Bold values are statistically significant.

**TABLE 3:** Multivariate analysis of prognostic factors.

	OS			PFS		
	HR	95% CI	*p*‐Value	HR	95% CI	*p*‐Value
Cycles of chemotherapy	0.6	0.410–0.835	**0.003**	0.6	0.436–0.872	**0.006**
KPS	0.9	0.913–0.970	**<0.001**	0.9	0.917–0.975	**<0.001**
Tumor stage	1.3	1.040–1.650	**0.022**	1.3	1.050–1.670	**0.018**
Tumor response after CCRT	1.4	1.080–1.910	**0.013**	1.2	0.919–1.610	0.170
PCI	0.6	0.437–0.811	**0.001**	0.6	0.419–0.765	**<0.001**

*Note*: Bold values are statistically significant.

Abbreviations: CCRT, concurrent chemo‐radiotherapy; CI, confidential interval; HR, hazard ratio; KPS, Karnofsky performance score; OS, overall survival; PCI, prophylactic cranial irradiation; PFS, progression free survival.

### 3.5 Toxicity

The major treatment‐related toxicities, encompassing both hematologic and non‐hematologic acute as well as late toxicities, are summarized in Table 4. Hematologic adverse events were generally of mild to moderate severity in both groups. Notably, there was no statistically significant disparity in hematological toxicity (grade ≥ 3) between the 6 cycles and 4–5 cycles cohorts (76.7% and 84.9%, respectively, with a *p*‐value of 0.195, before PSM). Likewise, there was no significant difference in acute radiation toxicities between the 6 cycles and 4–5 cycles groups. The most common acute radiation injuries (See Table S1 for details) in the 6 cycles group were grade 1 pneumonia (35.0%) and grade 1 esophagitis (60.0%), whereas in the 4–5 cycles group they were grade 1 pneumonia (42.0%) and grade 1 esophagitis (46.8%). Furthermore, late radiotherapy‐induced toxicity primarily manifested as mild to moderate lung and esophageal damage. Grade 2–3 lung injury was observed in 6 patients (10.0%) in the 6 cycles group and 13 patients (6.3%) in the 4–5 cycles group. The incidence of grade ≥1 sensory neuropathy after 6 cycles of treatment (21.7%) exceeded that of 4–5 cycles (2.0%) (*p* < 0.001). Late spinal cord toxicity was not observed.

**TABLE 4:** Treatment‐related toxicities of 4–5 cycles and 6 cycles groups.

	Before propensity score matching No. (%) (*n* = 265)			After propensity score matching No. (%) (*n* = 106)		
	4–5 cycles	6 cycles	*p*‐Value	4–5 cycles	6 cycles	*p*‐Value
	(*n* = 205)	(*n* = 60)		(*n* = 53)	(*n* = 53)	
Acute toxicity						
Hematologic			0.195			0.626
<3	31 (15.1%)	14 (23.3%)		9 (17.0%)	12 (22.6%)	
≥3	174 (84.9%)	46 (76.7%)		44 (83.0%)	41 (77.4%)	
Leucopenia			0.108			0.235
<3	107 (52.2%)	39 (65.0%)		28 (52.8%)	35 (66.0%)	
≥3	98 (47.8%)	21 (35.0%)		25 (47.2%)	18 (34.0%)	
Neuoenia			0.338			1.000
<3	75 (36.6%)	27 (45.0%)		23 (43.4%)	23 (43.4%)	
≥3	130 (63.4%)	33 (55.0%)		30 (56.6%)	30 (56.6%)	
Thrombocytopenia			1.000			1.000
<3	156 (76.1%)	46 (76.7%)		39 (73.6%)	40 (75.5%)	
≥3	49 (23.9%)	14 (23.3%)		14 (26.4%)	13 (24.5%)	
Anemia			0.355			0.383
<3	146 (71.2%)	47 (78.3%)		36 (67.9%)	41 (77.4%)	
≥3	59 (28.8%)	13 (21.7%)		17 (32.1%)	12 (22.6%)	
Pneumonitis			**0.030**			0.437
<2	201 (98.0%)	55 (91.7%)		51 (96.2%)	48 (90.6%)	
≥2	4 (2.0%)	5 (8.3%)		2 (3.8%)	5 (9.4%)	
Esophagitis			0.073			0.680
<2	122 (59.5%)	44 (73.3%)		34 (64.2%)	37 (69.8%)	
≥2	83 (40.5%)	16 (26.7%)		19 (35.8%)	16 (30.2%)	
Late toxicity						
Pulmonary injury			0.392			0.716
<2	192 (93.7%)	54 (90.0%)		50 (94.3%)	48 (90.6%)	
≥2	13 (6.3%)	6 (10.0%)		3 (5.7%)	5 (9.4%)	
Esophageal injury			0.737			1.000
<1	196 (95.6%)	57 (95.0%)		50 (94.3%)	50 (94.3%)	
≥1	9 (4.4%)	3 (5.00%)		3 (5.7%)	3 (5.7%)	
Neuropathy			**<0.001**			**0.010**
<1	201 (98.0%)	47 (78.3%)		51 (96.2%)	41 (77.4%)	
≥1	4 (2.0%)	13 (21.7%)		2 (3.8%)	12 (22.6%)	

*Note*: Bold values are statistically significant.

## 4 DISCUSSION

Platinum‐based chemotherapy, including etoposide and cisplatin, showed potential improvements in OS for SCLC.^7^ In LS‐SCLC patients, the guidelines recommend 4 to 6 cycles of platinum‐based chemotherapy using cisplatin or carboplatin in combination with etoposide, which is rated as grade 1A over other chemotherapy regimens.^8^ However, in the current era where the standard treatment for LS‐SCLC involves combining etoposide and platinum‐based chemotherapy with twice daily hyperfractionated thoracic radiotherapy, there was no study that compared the efficacy and side effects of different cycles (ranging from 4 to 6 cycles) of first‐line platinum‐based chemotherapy. Consequently, the optimal number of etoposide and platinum‐based chemotherapy cycles for LS‐SCLC remains unresolved.

In a previous study, we conducted a randomized trial to determine the optimal target volumes for concurrent TRT in patients with LS‐SCLC.^5^ The chemotherapy planned to be delivered in this trial consisted of 4–6 cycles of EP. By utilizing data from our previous prospective trial, we undertook a comparative analysis of the outcomes of 6 versus 4–5 cycles of EP regimen chemotherapy in patients with LS‐SCLC who underwent concurrent TRT. This analysis reported that the 5‐year OS rates were 29.87% and 15.72% in the 6 cycles and 4–5 cycles group (after PSM), respectively. Additionally, patients who completed 6 cycles of EP chemotherapy demonstrated significantly prolonged median OS and PFS. Currently, the NCCN guidelines recommend that SCLC patients should be treated with 4–6 cycles of standard chemotherapy. Those received with no less than 4 cycles of chemotherapy had exhibited higher median survival in LS‐SCLC patients.^9^ Additionally, a retrospective analysis conducted by Cao et al. involving 707 SCLC patients demonstrated that chemotherapy cycles≥4 was an independent positive prognostic factor for SCLC patients treated with EP‐based chemotherapy.^10^ Moreover, prolonging chemotherapy cycles beyond the standard 4 to 6 cycles leads to only a minor extension in response duration without survival benefits and brings a greater risk of side effects.^2^, ^4^ The definitive answer concerning the ideal number of platinum‐based chemotherapy cycles (ranging from 4 to 6) for LS‐SCLC patients has remained elusive due to the absence of randomized clinical trials. In the past 20 years, 4 cycles of first‐line EP‐based chemotherapy were widely used in most large randomized phase III trials for LS‐SCLC, such as Intergroup 0096,^11^ JCOG 9104,^12^ a prospective clinical trial from South Korea,^13^ JCOG0202.^14^ Some guidelines even recommend that 4 cycles be preferred in LS‐SCLC.^8^ Four cycles of chemotherapy were also recommended in a recent clinical study by Bogart J,^15^ Grønberg^16^ et al. This choice might be attributed to the potential cumulative toxicity arising from the combination of high‐intensity radiotherapy and chemotherapy.

However, in the CONVERT trial^17^ which was the phase 3 trial completed investigating thoracic radiotherapy in LS‐SCLC, chemotherapy contained 4–6 cycles of EP every 3 weeks. The average number of courses in the 45Gy group was 4.62, with 68% completing 4–5 cycles of chemotherapy and 21% completing 6 cycles. In another study of hypofractionated doses of SCLC, the average number of chemotherapy courses in the 45Gy group was 4.265, 14.9% received 6 cycles of chemotherapy, and 80.8% received 4–5 cycles of chemotherapy.^18^ Unfortunately, both of these trials have not reported patient data comparing the OS of patients who received 6 cycles versus 4–5 cycles of chemotherapy.

In 1998, Veslemes et al.^19^ conducted a randomized study comparing 4 versus 6 cycles of EP in small cell lung cancer, which was the first and the only prospective study in this field. The results indicated a trend in favor of the 6‐course group for patients with extensive‐stage small cell lung cancer (ES‐SCLC), leading to a MST of 9 months. In comparison, those in the 4‐course group showed a MST of 6.5 months (*p* = 0.09). Nevertheless, no statistically significant differences in survival were observed among patients with limited disease, with 10.5 months and 12 months in the 4‐course and 6‐course groups, respectively. It is noteworthy that the study comprised a total of 69 evaluable SCLC patients. Among these, only 10 patients with limited disease underwent 4 cycles of EP, while 13 patients with limited disease received 6 cycles of EP. Additionally, patients with LS‐SCLC received megavoltage radiotherapy to a midline dose of 42 Gy after completing the fourth course without receiving PCI. Hence, it was insufficient to draw definitive conclusions about the optimal duration of chemotherapy for LS‐SCLC from that study.

Several retrospective studies have also explored prognostic factors including the number of chemotherapy cycles in SCLC.^9^, ^10^, ^20^, ^21^, ^22^ However, most of these studies compared the survivals of <4 and ≥4 cycles of chemotherapy, and fewer studies compared the survivals within 4–6 cycles of chemotherapy. Hermes et al.^20^ conducted a review of 397 SCLC patients, and the results revealed that the number of chemotherapy cycles had a significant impact on MST for ES‐SCLC. However, there was no statistically significant difference in MST based on the number of cycles administered as first‐line chemotherapy for LS‐SCLC. In LS‐SCLC patients who received up to 4 cycles, the MST was of 18.5 months (*N* = 75, 61%), while it reached 18.7 months when they were given 5 or 6 cycles as initial therapy (*N* = 45, 39%). Scotti et al.^22^ reviewed 57 LS‐SCLC patients who underwent no less than 3 cycles of platinum‐based chemotherapy with TRT in the first cycle of EP. The results revealed that the number of chemotherapy cycles continues to be a substantial predictor of mortality. Patients who underwent 5–6 cycles of chemotherapy exhibited a reduced risk of death in contrast to those who received 3–4 cycles (HR 0.44).

Our study demonstrated the superiority of 6 cycles of standard EP treatment in enhancing the survival of LS‐SCLC patients when compared with 4 or 5 cycles, and 6 cycles were independent favorable prognostic factors for OS and PFS. Thus, for patients who can tolerate the treatment well and show no evidence of disease progression, continuing chemotherapy for up to 6 cycles may be the preferred option. Fewer than 6 cycles of chemotherapy might not be enough for patients who can tolerate the treatment well. At the same time, these findings suggest the potential significance of altering chemotherapy cycles, although further investigations are imperative to substantiate these conclusions. These results hold significant implications for the optimal administration of EP‐based chemotherapy in LS‐SCLC patients. Patients who tolerate the chemotherapy regimen well stand to reap the most substantial survival benefit by adhering to a six‐cycle treatment course, until disease progression occurs or unacceptable adverse effects dictate otherwise.

Immunotherapy, as an emerging treatment method, has a remarkable effect in the treatment of ES‐SCLC^23^, ^24^ and has increasingly been explored for its potential in LS‐SCLC management in recent years. Welsh conducted a phase 1/2 trial combining CCRT with pembrolizumab, revealing a regimen characterized by favorable tolerability and outcomes. Notably, the trial showed a median PFS of 19.7 months and a median OS of 39.5 months.^25^ However, the STIMULI study showed that the combination of nivolumab and ipilimumab in LS‐SCLC after standard treatment did not achieve the endpoint of improving PFS but increased immunotherapy‐related toxicity.^26^ Chemotherapy combined with immunotherapy stands as the favored treatment for ES‐SCLC; however, its application in LS‐SCLC remains a subject of debate, necessitating additional clinical research evidence.

### 4.1 Limitations

An obvious limitation of the study lies in its retrospective nature, even though the data was from our previous prospective randomized study. Moreover, the chemotherapy cycles administered to LS‐SCLC patients depended on different medical oncologists, whose decisions on the number of chemotherapy cycles could have been influenced by an array of factors, including the side effects of previous chemotherapy, performance status after CCRT, the compliance of patients, and financial condition of patients. Although the exclusion of patients who discontinued treatment owing to disease progression or received four or fewer cycles of chemotherapy for various reasons aimed to mitigate the impact of confounding factors. It is important to note that despite adjustments made in the multivariate analysis, these issues may still have potentially confounded or biased our primary findings. Furthermore, the sample size of patients in the 6 cycles chemotherapy group is relatively small. It could be attributed to the fact that most patients may not have been able to tolerate 6 cycles of chemotherapy, particularly due to the occurrence of severe adverse effects such as gastrointestinal irritation and bone marrow suppression after CCRT. Hence, in the context of modern radiotherapy techniques and supportive care, it is essential to conduct a prospective study that directly compares the benefits of continuing etoposide and platinum‐based chemotherapy for 6 cycles versus 4 or 5 cycles.

## 5 CONCLUSION

Compared to 4 or 5 cycles, administering 6 cycles of standard chemotherapy markedly enhanced both the OS and PFS in LS‐SCLC patients undergoing a regimen of EP chemotherapy combined with twice daily hyperfractionated TRT. The conduction of a prospective study incorporating modern radiotherapy techniques and comprehensive supportive care holds promise in furnishing additional compelling evidence in favor of this approach.

## AUTHOR CONTRIBUTIONS

**Tian‐tian Yu:** Conceptualization (equal); data curation (equal); formal analysis (equal); investigation (equal); methodology (equal); validation (equal); writing – original draft (equal). **Xiao Hu:** Conceptualization (equal); data curation (equal); formal analysis (equal); investigation (equal); methodology (equal); software (equal); supervision (equal); validation (equal). **Wei‐jian Liufu:** Data curation (equal); formal analysis (equal); investigation (equal); methodology (equal); software (equal). **Shao‐qing Niu:** Formal analysis (equal); investigation (equal). **Hui‐min Lian:** Formal analysis (equal); investigation (equal). **Hong‐lian Ma:** Formal analysis (equal); investigation (equal). **Jin Wang:** Formal analysis (equal); investigation (equal). **Yong Bao:** Conceptualization (equal); data curation (equal); formal analysis (equal); funding acquisition (equal); investigation (equal); methodology (equal); project administration (equal); supervision (equal); validation (equal); writing – review and editing (equal). **Ming Chen:** Conceptualization (equal); data curation (equal); formal analysis (equal); funding acquisition (equal); investigation (equal); methodology (equal); project administration (equal); resources (equal); supervision (equal); validation (equal); visualization (equal); writing – review and editing (equal). **Fang Peng:** Conceptualization (equal); data curation (equal); formal analysis (equal); funding acquisition (equal); investigation (equal); methodology (equal); project administration (equal); resources (equal); supervision (equal); writing – original draft (equal); writing – review and editing (equal).

## FUNDING INFORMATION

This work was funded by the National Natural Science Foundation of China (No. 81602661, No. 82272744), the Natural Science Foundation of Guangdong Province, China (No. 2016A030310164, No. 2022A1515010814) and Beijing Xisike Clinical Oncology Research Foundation (No. 2019051). The funders played no role in study design, data collection and analysis, decision‐making regarding publication, or manuscript preparation.

## CONFLICT OF INTEREST STATEMENT

The authors affirm that there are no interests involved in this study.

## ETHICS STATEMENT

This trial was conducted in accordance with the principles outlined in the Helsinki Declaration and adhered to the guidelines of Good Clinical Practice. It received approval from the Clinical Ethics Committee of Sun‐Yat Sen University prior to the commencement of the study. The trial has been registered on ClinicalTrials.gov under the identifier NCT01731548.

## PATIENT CONSENT

Informed consent was obtained for all participants.

## Supporting information


Appendix S1.

## Data Availability

The dataset utilized and analyzed in the current investigation is not publicly accessible owing to the sensitivity of the data. It may be available by reaching out to the corresponding authors with a justifiable inquiry.

## References

[1] van Meerbeeck JP , Fennell DA , de Ruysscher DK . Small‐cell lung cancer. Lancet. 2011;378(9804):1741‐1755. 21565397 10.1016/S0140-6736(11)60165-7

[2] Kalemkerian GP , Akerley W , Bogner P , et al. Small cell lung cancer. J Natl Compr Canc Netw. 2013;11(1):78‐98. 23307984 10.6004/jnccn.2013.0011 PMC3715060

[3] Zhou H , Zeng C , Wei Y , et al. Duration of chemotherapy for small cell lung cancer: a meta‐analysis. PLoS One. 2013;8(8):e73805. 24023692 10.1371/journal.pone.0073805 PMC3758337

[4] Jh S , Adak S , Cella D , et al. Topotecan versus observation after cisplatin plus etoposide in extensive‐stage small‐cell lung cancer: E7593—a phase III trial of the Eastern Cooperative Oncology Group. J Clin Oncol. 2001;19(8):2114‐2122. 11304763 10.1200/JCO.2001.19.8.2114

[5] Hu X , Bao Y , Zhang L , et al. Omitting elective nodal irradiation and irradiating postinduction versus preinduction chemotherapy tumor extent for limited‐stage small cell lung cancer: interim analysis of a prospective randomized noninferiority trial. Cancer. 2012;118(1):278‐287. 21598237 10.1002/cncr.26119

[6] Hu X , Bao Y , Yj X , et al. Final report of a prospective randomized study on thoracic radiotherapy target volume for limited‐stage small cell lung cancer with radiation dosimetric analyses. Cancer. 2020;126(4):840‐849. 31714592 10.1002/cncr.32586

[7] Amini A , La B , Jw W , et al. Progress in the management of limited‐stage small cell lung cancer. Cancer. 2014;120(6):790‐798. 24327434 10.1002/cncr.28505 PMC3947683

[8] Cm R , Ismaila N , Cl H , et al. Treatment of small‐cell lung cancer: American Society of Clinical Oncology endorsement of the American College of Chest Physicians Guideline. J Clin Oncol. 2015;33(34):4106‐4111. 26351333 10.1200/JCO.2015.63.7918

[9] Wen Q , Meng X , Xie P , et al. Evaluation of factors associated with platinum‐sensitivity status and survival in limited‐stage small cell lung cancer patients treated with chemoradiotherapy. Oncotarget. 2017;8(46):81405‐81418. 29113400 10.18632/oncotarget.19073 PMC5655295

[10] Cao S , Jin S , Shen J , et al. Selected patients can benefit more from the management of etoposide and platinum‐based chemotherapy and thoracic irradiation‐a retrospective analysis of 707 small cell lung cancer patients. Oncotarget. 2017;8(5):8657‐8669. 28055965 10.18632/oncotarget.14395 PMC5352430

[11] At T 3rd , Kim K , Blum R , et al. Twice‐daily compared with once‐daily thoracic radiotherapy in limited small‐cell lung cancer treated concurrently with cisplatin and etoposide. New Engl J Med. 1999;340(4):265‐271. 9920950 10.1056/NEJM199901283400403

[12] Takada M , Fukuoka M , Kawahara M , et al. Phase III study of concurrent versus sequential thoracic radiotherapy in combination with cisplatin and etoposide for limited‐stage small‐cell lung cancer: results of the Japan clinical oncology group study 9104. J Clin Oncol. 2002;20(14):3054‐3060. 12118018 10.1200/JCO.2002.12.071

[13] Jm S , Yc A , Ek C , et al. Phase III trial of concurrent thoracic radiotherapy with either first‐ or third‐cycle chemotherapy for limited‐disease small‐cell lung cancer. Ann Oncol. 2013;24(8):2088‐2092. 23592701 10.1093/annonc/mdt140

[14] Kubota K , Hida T , Ishikura S , et al. Etoposide and cisplatin versus irinotecan and cisplatin in patients with limited‐stage small‐cell lung cancer treated with etoposide and cisplatin plus concurrent accelerated hyperfractionated thoracic radiotherapy (JCOG0202): a randomised phase 3 study. Lancet Oncol. 2014;15(1):106‐113. 24309370 10.1016/S1470-2045(13)70511-4

[15] Bogart J , Wang X , Masters G , et al. High‐dose once‐daily thoracic radiotherapy in limited‐stage small‐cell lung cancer: CALGB 30610 (Alliance)/RTOG 0538. J Clin Oncol. 2023;41(13):2394‐2402. 36623230 10.1200/JCO.22.01359 PMC10150922

[16] Bh G , Kt K , Fløtten Ø , et al. High‐dose versus standard‐dose twice‐daily thoracic radiotherapy for patients with limited stage small‐cell lung cancer: an open‐label, randomised, phase 2 trial. Lancet Oncol. 2021;22(3):321‐331. 33662285 10.1016/S1470-2045(20)30742-7

[17] Faivre‐Finn C , Snee M , Ashcroft L , et al. Concurrent once‐daily versus twice‐daily chemoradiotherapy in patients with limited‐stage small‐cell lung cancer (CONVERT): an open‐label, phase 3, randomised, superiority trial. Lancet Oncol. 2017;18(8):1116‐1125. 28642008 10.1016/S1470-2045(17)30318-2 PMC5555437

[18] Qiu B , Li Q , Liu J , et al. Moderately Hypofractionated once‐daily compared with twice‐daily thoracic radiation therapy concurrently with etoposide and cisplatin in limited‐stage small cell lung cancer: a multicenter, phase II, randomized trial. Int J Radiat Oncol Biol Phys. 2021;111(2):424‐435. 33992717 10.1016/j.ijrobp.2021.05.003

[19] Veslemes M , Polyzos A , Latsi P , et al. Optimal duration of chemotherapy in small cell lung cancer: a randomized study of 4 versus 6 cycles of cisplatin‐etoposide. J Chemother. 1998;10(2):136‐140. 9603640 10.1179/joc.1998.10.2.136

[20] Hermes A , Waschki B , Gatzemeier U , et al. Characteristics, treatment patterns and outcomes of patients with small cell lung cancer—a retrospective single institution analysis. Lung Cancer. 2011;71(3):363‐366. 20619477 10.1016/j.lungcan.2010.06.003

[21] Liu S , Guo H , Kong L , et al. The prognostic factors in the elderly patients with small cell lung cancer: a retrospective analysis from a single cancer institute. Int J Clin Exp Pathol. 2015;8(9):11033‐11041. 26617821 PMC4637636

[22] Scotti V , Meattini I , Saieva C , et al. Limited‐stage small‐cell lung cancer treated with early chemo‐radiotherapy: the impact of effective chemotherapy. Tumori. 2012;98(1):53‐59. 22495702 10.1177/030089161209800107

[23] Paz‐Ares L , Dvorkin M , Chen Y , et al. Durvalumab plus platinum‐etoposide versus platinum‐etoposide in first‐line treatment of extensive‐stage small‐cell lung cancer (CASPIAN): a randomised, controlled, open‐label, phase 3 trial. Lancet. 2019;394(10212):1929‐1939. 31590988 10.1016/S0140-6736(19)32222-6

[24] Wang J , Zhou C , Yao W , et al. Adebrelimab or placebo plus carboplatin and etoposide as first‐line treatment for extensive‐stage small‐cell lung cancer (CAPSTONE‐1): a multicentre, randomised, double‐blind, placebo‐controlled, phase 3 trial. Lancet Oncol. 2022;23(6):739‐747. 35576956 10.1016/S1470-2045(22)00224-8

[25] Jw W , Jv H , Guo C , et al. Phase 1/2 trial of pembrolizumab and concurrent chemoradiation therapy for limited‐stage SCLC. J Thorac Oncol. 2020;15(12):1919‐1927. 32916308 10.1016/j.jtho.2020.08.022 PMC10600713

[26] Peters S , Jl P , Dafni U , et al. Consolidation nivolumab and ipilimumab versus observation in limited‐disease small‐cell lung cancer after chemo‐radiotherapy – results from the randomised phase II ETOP/IFCT 4‐12 STIMULI trial. Ann Oncol. 2022;33(1):67‐79. 34562610 10.1016/j.annonc.2021.09.011

