# Supplementary material for: Six versus four or five cycles of first‐line etoposide and platinum‐based chemotherapy combined with thoracic radiotherapy in patients with limited‐stage small‐cell lung cancer: A propensity score‐matched analysis of a prospective randomized trial
Source: Cancer Med. 2024 Apr 25;13(8):e7215. doi: 10.1002/cam4.7215 (PMC11043670; doi:10.1002/cam4.7215)
Supplement: Supplementary file 1 — Appendix S1. [file CAM4-13-e7215-s001.docx]

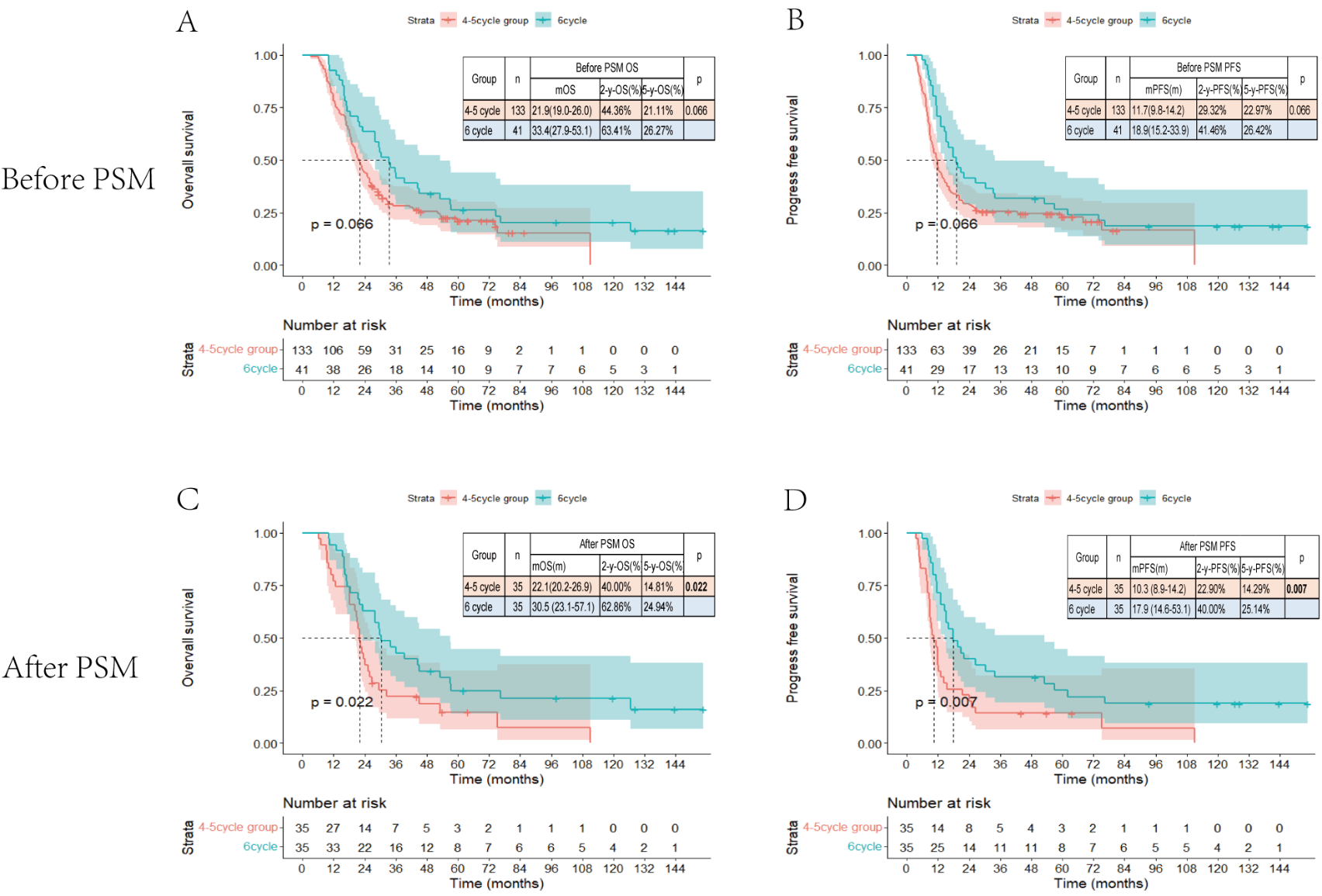
**Figure S1 OS and PFS curves for after CCRT who achieved PR.**

(A) Before PSM, OS for patients in 4-5 cycles and 6 cycles group (n = 174, P =0.066); (B) Before PSM, PFS for patients in 4-5 cycles and 6 cycles group (n = 174, P =0.066). (C)After PSM, OS for patients in 4-5 cycles and 6 cycles group (n = 70, P =0.022); (D)After PSM, PFS for patients in 4-5 cycles and 6 cycles group (n = 70, P =0.007).

**Figure S2 OS and PFS curves for cycle 4 and cycle 5**


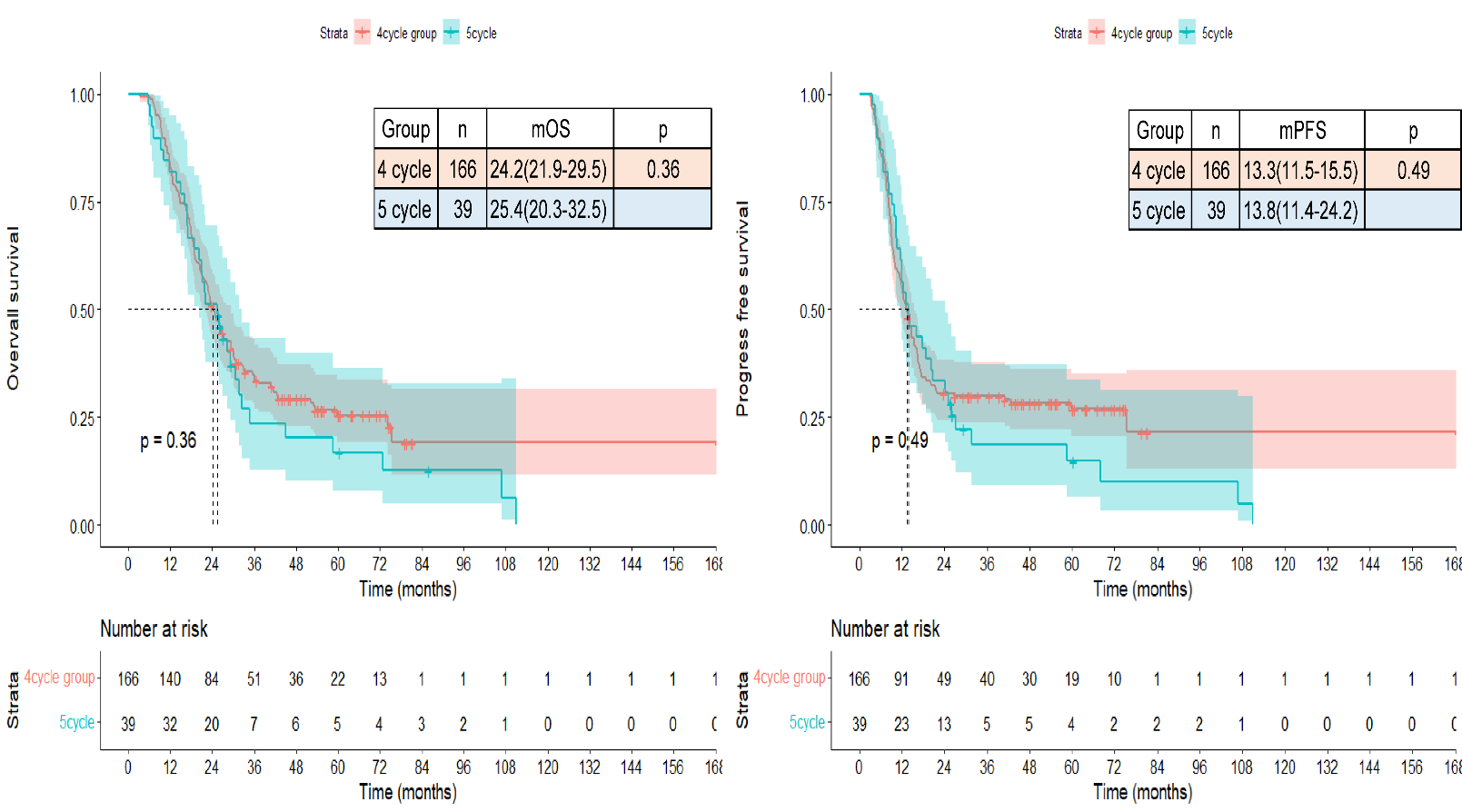


B

A

(A) OS for patients in 4 cycles and 5 cycles group (n = 205, P =0.36); (B) PFS for patients in 4 cycles and 5 cycles group (n = 205, P =0.49).

Table S1.The treatment-related toxicities

|  | Before propensity score matching No. (%) (n = 265) | | |  | After propensity score matching No. (%) (n = 106) | | |
| --- | --- | --- | --- | --- | --- | --- | --- |
|  | 4-5 cycles | 6 cycles | *P-value* |  | 4-5 cycles | 6 cycles | *P-value* |
|  | (n=205) | (n=60) |  |  | (n=53) | (n=53) |  |
| **Acute toxity** |  |  |  |  |  |  |  |
| Hematologic: |  |  | 0.242 |  |  |  | 0.734 |
| 0 | 0 (0.0%) | 1 (1.7%) |  |  | 0 (0.0%) | 0 (0.0%) |  |
| 1 | 5 (2.4%) | 2 (3.3%) |  |  | 1 (1.9%) | 2 (3.8%) |  |
| 2 | 26 (12.7%) | 11 (18.3%) |  |  | 8 (15.1%) | 10 (18.9%) |  |
| 3 | 103 (50.2%) | 30 (50.0%) |  |  | 25 (47.2%) | 27 (50.9%) |  |
| 4 | 71 (34.6%) | 16 (26.7%) |  |  | 19 (35.8%) | 14 (26.4%) |  |
| ≥3 | 174 (84.9%) | 46 (76.7%) |  |  | 44 (83.0%) | 41 (77.4%) |  |
| Leucopenia: |  |  | **0.004** |  |  |  | **0.014** |
| 0 | 5 (2.4%) | 7 (11.7%) |  |  | 1 (1.9%) | 5 (9.4%) |  |
| 1 | 16 (7.8%) | 11 (18.3%) |  |  | 1 (1.9%) | 10 (18.9%) |  |
| 2 | 86 (42.0%) | 21 (35.0%) |  |  | 26 (49.1%) | 20 (37.7%) |  |
| 3 | 83(40.5%) | 17 (28.3%) |  |  | 19 (35.8%) | 14 (26.4%) |  |
| 4 | 15 (7.3%) | 4 (6.7%) |  |  | 6 (11.3%) | 4 (7.6%) |  |
| ≥3 | 98 (47.8%) | 21 (35.0%) |  |  | 25(47.2%) | 18 (34.0%) |  |
| Neuoenia: |  |  | 0.184 |  |  |  | 0.062 |
| 0 | 9 (4.4%) | 6 (10.0%) |  |  | 3 (5.7%) | 4 (7.6%) |  |
| 1 | 13 (6.3%) | 8 (13.3%) |  |  | 1 (1.9%) | 8 (15.1%) |  |
| 2 | 53 (25.9%) | 13 (21.7%) |  |  | 19 (35.8%) | 11 (20.8%) |  |
| 3 | 80 (39.0%) | 20 (33.3%) |  |  | 15 (28.3%) | 19 (35.8%) |  |
| 4 | 50 (24.4%) | 13 (21.7%) |  |  | 15 (28.3%) | 11 (20.8%) |  |
| ≥3 | 130 (63.4%) | 33 (55.0%) |  |  | 30 (56.6%) | 30 (56.6%) |  |
| Thrombocytopenia: |  |  | 0.247 |  |  |  | 0.39 |
| 0 | 83 (40.5%) | 33 (55.0%) |  |  | 18 (34.0%) | 27 (50.9%) |  |
| 1 | 37 (18.0%) | 6 (10.0%) |  |  | 12 (22.6%) | 6 (11.3%) |  |
| 2 | 36 (17.6%) | 7 (11.7%) |  |  | 9 (17.0%) | 7 (13.2%) |  |
| 3 | 33 (16.1%) | 8 (13.3%) |  |  | 8 (15.1%) | 7 (13.2%) |  |
| 4 | 16 (7.8%) | 6 (10.0%) |  |  | 6 (11.3%) | 6 (11.3%) |  |
| ≥3 | 49 (23.9%) | 14 (23.3%) |  |  | 14 (26.4%) | 13 (24.5%) |  |
| Anemia: |  |  | 0.623 |  |  |  | 0.853 |
| 0 | 12 (5.9%) | 5 (8.3%) |  |  | 4 (7.6%) | 4 (7.6%) |  |
| 1 | 44 (21.5%) | 15 (25.0%) |  |  | 11 (20.8%) | 12 (22.6%) |  |
| 2 | 90 (43.9%) | 27 (45.0%) |  |  | 21 (39.6%) | 25 (47.2%) |  |
| 3 | 41 (20.0%) | 11 (18.3%) |  |  | 13 (24.5%) | 10 (18.9%) |  |
| 4 | 18 (8.8%) | 2 (3.3%) |  |  | 4 (7.6%) | 2 (3.8%) |  |
| ≥3 | 59 (28.8%) | 13 (21.7%) |  |  | 17(32.1%) | 12 (22.6%) |  |
| Pneumonitis: |  |  | 0.086 |  |  |  | 0.593 |
| 0 | 115 (56.1%) | 34 (56.7%) |  |  | 29 (54.7%) | 30 (56.6%) |  |
| 1 | 86 (42.0%) | 21 (35.0%) |  |  | 22 (41.5%) | 18 (34.0%) |  |
| 2 | 3 (1.5%) | 4 (6.7%) |  |  | 2 (3.8%) | 4 (7.6%) |  |
| 3 | 1 (0.5%) | 1 (1.7%) |  |  | 0 (0.0%) | 1 (1.9%) |  |
| ≥2 | 4（2.0%） | 5 (8.3%） |  |  | 2 (3.8%) | 5 (9.4%) |  |
| Esophagitis: |  |  | 0.153 |  |  |  | 0.872 |
| 0 | 26 (12.7%) | 8 (13.3%) |  |  | 7 (13.2%) | 7 (13.2%) |  |
| 1 | 96 (46.8%) | 36 (60.0%) |  |  | 27 (50.9%) | 30 (56.6%) |  |
| 2 | 60 (29.3%) | 14 (23.3%) |  |  | 15 (28.3%) | 14 (26.4%) |  |
| 3 | 23 (11.2%) | 2 (3.3%) |  |  | 4 (7.6%) | 2 (3.8%) |  |
| ≥2 | 83（40.5%） | 16 (26.7%） | |  | 19 (35.8%) | 16(30.2%) |  |
| **Late toxicity** |  |  |  |  |  |  |  |
| Pulmonary injury: |  |  | 0.076 |  |  |  | 0.071 |
| 0 | 152 (74.1%) | 35 (58.3%) |  |  | 42 (79.2%) | 32 (60.4%) |  |
| 1 | 40 (19.5%) | 19 (31.7%) |  |  | 8 (15.1%) | 16 (30.2%) |  |
| 2 | 12 (5.9%) | 5 (8.3%) |  |  | 2 (3.8%) | 5 (9.4%) |  |
| 3 | 1 (0.5%) | 1 (1.7%) |  |  | 1 (1.9%) | 0 (0.0%) |  |
| ≥2 | 13(6.3%) | 6 (10.0%) |  |  | 3 (5.7%) | 5 (9.4%) |  |
| Esophageal injury: |  |  | 0.329 |  |  |  | 1 |
| 0 | 196 (95.6%) | 57 (95.0%) |  |  | 50 (94.3%) | 50 (94.3%) |  |
| 1 | 9 (4.4%) | 2 (3.3%) |  |  | 3 (5.7%) | 2 (3.8%) |  |
| 2 | 0 (0.0%) | 1 (1.7%) |  |  | 0 (0.0%) | 1 (1.9%) |  |
| ≥1 | 9 (4.4%） | 3 (5.0%) |  |  | 3 (5.7%) | 3 (5.7%) |  |
| Neuropathy : |  |  | **<0.001** |  |  |  | **0.01** |
| 0 | 201 (98.0%) | 47 (78.3%) |  |  | 51 (96.2%) | 41 (77.4%) |  |
| 1 | 4 (2.0%) | 13 (21.7%) |  |  | 2 (3.8%) | 12 (22.6%) |  |

Bold values are statistically significant.
